# Increased frequency of circulating Th22 in addition to Th17 and Th2 lymphocytes in systemic sclerosis: association with interstitial lung disease

**DOI:** 10.1186/ar3486

**Published:** 2011-10-13

**Authors:** Marie-Elise Truchetet, Nicolò C Brembilla, Elisa Montanari, Yannick Allanore, Carlo Chizzolini

**Affiliations:** 1 Immunology and Allergy, University Hospital and School of Medicine, 4 rue Gabrielle Perret Gentil, Geneva 1211, Switzerland; 2 Service de Rhumatologie A, Hôpital Cochin, Paris Descartes University, 27 rue du Faubourg Saint-Jacques, Paris 75679, France; 3 INSERM U1016, Institut Cochin, Sorbonne Paris Cité, Paris Descartes University, 27 rue du Faubourg Saint-Jacques, Paris 75014, France

## Abstract

**Introduction:**

T cell abnormalities have been associated with the pathogenesis of systemic sclerosis (SSc). Recently, besides T helper (Th)17 cells, the Th22 subset has been identified in humans. Our purpose was to investigate the pattern of cytokines produced and chemokine-receptors expressed by peripheral blood (PB) Th cells in SSc and healthy donors (HD) focusing on cells producing interleukin (IL)-17 and IL-22 and to identify specific clinical associations.

**Methods:**

Clinical data and peripheral blood were collected in 33 SSc individuals and 29 HD. IL-17A, IL-22, interferon gamma (IFN-γ), IL-4 production, the chemokine receptors CCR4, CCR6, CCR10, CXCR3 expression and the CD161 Th17 cell marker were assessed by multiparametric flow cytometry in PB CD4+ T cells. Intracellular cytokine accumulation was further investigated in CD4+ T cells expanded *in vitro *for seven days.

**Results:**

The frequency of Th22, Th17, Th2, but not Th1 cells, was significantly increased in SSc individuals compared to HD. The percentage of CD161+CD4+ T cells was increased in SSc and correlated with the percentage of IL-17A producing cells. Moreover, the expression of the skin- and lung-homing chemokine receptor CCR6 correlated with the frequency of IL-22 and IL-17A-producing cells in SSc but not in HD. Finally, SSc interstitial lung disease (ILD) was strongly associated with higher numbers of IL-22 and, to a lesser extent, IL-17A-producing cells.

**Conclusions:**

IL-22 and IL-17A-producing T cells with skin- and lung-homing capabilities are characteristically increased in SSc. These findings support the hypothesis that Th22, in addition to Th17 cells, may be involved in pathological processes leading to SSc. While the association between IL-22 producing cells and ILD needs to be assessed in larger cohorts of patients, the increased frequency of Th22 cells appears to be a useful novel biomarker in SSc.

## Introduction

Systemic sclerosis (SSc), or scleroderma, is a chronic connective tissue disease characterized by autoimmunity, fibrosis of the skin and internal organs, and vascular dysfunction [1]. While the pathogenic mechanisms of the disease are still largely elusive, a number of findings indicate that the immune response may play a key role [2]. First, antinuclear antibodies (ANA) are characteristically present and segregate with distinct clinical presentations. Second, genetic studies indicate that most of the gene polymorphisms associated with SSc involve genes coding for molecules controlling the immune response, shared with other auto-immune disorders like systemic lupus erythematosus (SLE) [3]. Third, histological examination of the skin of patients with SSc during the early edematous inflammatory phase of the disease demonstrates the presence of mononuclear cell infiltrates containing T cells with perivascular distribution preceding the development of fibrosis and overt vasculopathy [4,5]. Of interest, fibroblasts with increased expression of type I and III procollagen mRNA are frequently detected in areas adjacent to mononuclear cell infiltrates suggesting that inflammatory cells, and in particular T cells, are responsible for the altered functional fibroblast phenotype [6].

Chemokine receptors sense the appropriate ligands in the extracellular environment and transduce the signal directing cell movement [7]. Importantly, in conjunction with adhesion molecules, they determine the combinatorial code used by immune cells to transmigrate across the endothelium and reach target tissues, both in homeostatic and inflamed conditions [8]. CCR4, and to a more limited degree CCR10, contribute to homing into the skin [9-11]. CCR6 has been shown to allow homing into the skin and other tissues including the lung, particularly under inflammatory conditions [11-13]. CD4+ T cells differentiate into a variety of effector subsets, which include T helper (Th)1, Th2, Th17, and the more recently identified Th22 cells [14,15]. Of interest, chemokine receptor distribution is characteristically restricted to discrete Th cell subsets [13,16-18]. Th1 cells mainly produce interferon gamma (IFN-γ) and are thought to preferentially express CXCR3 [16]. Th2 cells produce interleukin (IL)-4 and when activated preferentially express CCR4 [19]. Th17 cells produce IL-17A, IL-17F and IL-22 and mostly express CCR6 [13]. Moreover, Th17 cells have been recently shown to express the lectin receptor CD161, previously known as a natural killer cell marker [20,21]. IL-22 is produced by many cell types, including Th17 cells and Th1, while Th22 cells characteristically produce IL-22 in the absence of IL-17A and IFN-γ. Of interest, Th22 are enriched in cells expressing CCR4, CCR6, and CCR10 [17,18].

Th2 cells have been shown to be overrepresented in SSc tissues and to be linked to active disease compared to Th1 cells, since IL-4 has direct pro-fibrotic properties [22-25]. While Th17 cells are thought to play an important role in the induction of autoimmune tissue injury [26], little is known about their role in SSc. However, increased levels of IL-17 were detected in the sera and bronchoalveolar lavage fluid of SSc individuals [27,28], and in a recent study Th17 cells were found to be increased, especially in patients with early diffuse SSc [29]. As far as we know, no studies have yet directly assessed the presence and the functional characteristics of Th22 cells in SSc.

The objective of the present study was to revisit the contribution of various CD4+ T cell subsets to the peripheral cell pool characterizing SSc with major focus on cells producing IL-22 and IL-17A. We studied the chemokine receptor usage for assessing their potential to transmigrate into SSc affected tissues, and verified whether Th cell characteristics distinguishing SSc from healthy individuals (HD) could associate with specific SSc clinical features. The results indicate an SSc-specific increase in the number of Th cells producing IL-22 and IL-17A, skewed to preferential homing into the lung and associated with interstitial lung disease (ILD).

## Materials and methods

### Study population

The peripheral blood of 33 consecutive SSc individuals that satisfied the criteria by LeRoy *et al*. [30] and who were not receiving disease-modifying drugs, cytokine blocking reagents or immunosuppressant agents were prospectively recruited when presenting at the Rheumatology A Unit of the Cochin hospital during a nine- month period. Their clinical characteristics are detailed in Table 1. ILD was identified by the presence of typical features on high-resolution computerized tomography (HRCT) of the chest and confirmed by total lung capacity (TLC) lower than 80% of the predicted value. Pulmonary artery hypertension (PAH) was suggested by an echocardiographic systolic pulmonary arterial pressure > 40 mmHg, or a DLCO < 50% predicted in the absence of pulmonary fibrosis or unexplained dyspnea and confirmed by right heart catheterization and was defined as a mean resting pulmonary artery pressure > 25 mmHg in the presence of a pulmonary capillary wedge pressure £15 mmHg at right heart catheterization. Skin scores were not available and cutaneous involvement was expressed as limited (lSSc) or diffuse (dSSc) [30]. None of the recruited individuals was under treatment with immunosuppressant agents at the time of blood sampling. Peripheral blood from 29 age- and sex-matched HD was provided by the Blood Transfusion Center (Geneva University Hospital, Switzerland) (Table 1). This study was approved by the ethical committees of the institutions involved and was conducted according to the Declaration of Helsinki. Written informed consent was obtained from all individuals.

**Table 1:** Clinical characteristics of the study populations

Clinical and biological features	SSc (n: 33)	HD (n: 29)	*P*
Women, n (%)	29 (87.9)	23 (79.3)	ns
Age, median (range)	52 (30 to 80)	50 (18 to 68)	ns
Ethnicity: Caucasian (%)	30 (90)	29 (100)	ns
Diffuse cutaneous SSc, n (%)	20 (60.6)	N/A	
Age onset, mean (+/- SD)	44 (14)	N/A	
Disease duration, median (range)	7 (1 to 32)	N/A	
ANA positivity (%)	29 (87.9)	N/A	
ACA positivity (%)	4 (12.1)	N/A	
Anti-Scl70 positivity (%)	11 (33.3)	N/A	
Digital ulcers	19 (57.6)	N/A	
Calcinosis	9 (27.3)	N/A	
Arthritis	7 (21.2)	N/A	
PAH, n (%)	4 (12.1)	N/A	
ILD, n (%)	14 (42.4)	N/A	
Current immunosuppressive therapy (%)	0	N/A	

ANA, anti-nuclear antibodies; ACA, anti-centromere antibodies; ILD, interstitial lung disease; PAH, pulmonary artery hypertension; N/A: not applicable. *P *as determined by the Fisher's exact test. ILD was observed on HRCT and confirmed by a TLC < 80% of the predicted value. Pre-capillary PAH was defined as a mean resting pulmonary artery pressure > 25 mmHg in presence of a pulmonary capillary wedge pressure £15 mmHg by a right heart catheterization.

### Reagents

Anti-CD3 (clone OKT3) monoclonal antibody (mAb) was obtained from American Tissue Culture Collection (Manassas, VA, USA). Anti-CD4-APC-Cy7 (clone IVT114), anti-CD45-RA-FITC (clone HI100), anti-CCR6-PerCP-Cy5.5 (clone 11A9), anti-CCR4-PE-Cy7 (clone 1G1), anti-CXCR3-APC (clone 1C6/CXCR3), anti-CD161-APC (clone DX12) and anti-CD28 (clone CD28.2) mAbs came from BD Biosciences (San Jose, CA, USA); anti-IL-4-APC (clone 8D4-8 anti-IFN-γ-PE-Cy7 (clone 4S.B3) and anti-IL-17A-FITC (clone BL168) mAbs were from Biolegend (San Diego, CA, USA); anti-IL-22-PE (clone 142928), anti-CCR10-PE (clone 314305) came from R&D Systems (Abingdon, UK). The Cytofix/Cytoperm fixation/permeabilization solution kit was from Becton Dickinson (San Diego, CA, USA), Ficoll-Paque Plus from GE Healthcare (Uppsala, Sweden), RPMI 1640, phosphate buffered saline (PBS), glutamine, penicillin, streptomycin, trypsin, and fetal calf serum (FCS) from Gibco (Paisley, UK), and phorbol myristate acetate (PMA), β-mercaptoethanol and brefeldin A from Sigma (St. Louis, MO, USA). Human T-activator CD3/CD28 beads were obtained from Invitrogen (Oslo, Norway) and rhIL-2 from Biogen (Cambridge, MA, USA).

### Peripheral blood mononuclear cells (PBMC) culture conditions

PBMC were cryopreserved in liquid nitrogen until use, then thawed and maintained at 37°C for 16 h in RPMI 1640 supplemented with 1% nonessential amino acids, 1% L-glutamine, 1% sodium pyruvate, 50 U/ml penicillin, 50 μg/ml streptomycin, 5% pooled human AB serum, 5% FCS and 50 μM β-mercaptoethanol (complete medium) before use. For intracellular cytokine determination, PBMC were either cultured for 24 hours or 7 days upon activation by CD3/CD28 cross-linking. In a 24-hour assay, brefeldin A was added for the last 20 hours. In 7-day cultures IL-2 (20 U/ml) was added at 48 hours and the cells were re-stimulated for FACS analysis with PMA/ionomycin for the last 4.5 hours, in the presence of brefeldin A.

### Flow cytometry analysis

Surface staining was performed using fluorochrome-conjugated anti-CD4, anti-CD45RA, anti-CD161, anti-CCR6, anti-CCR4, anti-CXCR3 and anti-CCR10. In intracellular cytokine determination, the cells were stained with anti-CD4 mAb, fixed and incubated with anti-IL-17A, IL-22, IFNγ and IL-4 mAbs using a BD Cytofix/Cytoperm kit according to the manufacturer's instruction. FACS analysis was performed on FACSCanto flow cytometer using FACSDiva (Becton Dickinson) and FlowJo softwares (Tree Star Inc. Ashland, OR, USA). Irrelevant isotype-matched control mAb were used to determine specific staining.

### Statistical analysis

All populations satisfied the Kolmogorov-Smirnov normality test according to GraphPad Prism version 4.00 (Graphpad Software, La Jolla, CA, USA). The significant difference between samples was computed using the Student's *t *test and correlation between variables using the Pearson correlation coefficient. A *P-*value < 0.05 was considered statistically significant. Box plots were automatically generated using GraphPad. The box represents values between 25^th ^and 75^th ^percentile with a line at the median (50^th ^percentile). The whiskers extend above and below the box to show the values at the10^th ^and 90^th ^percentiles.

## Results

### Increased frequency of CD4+ T cells producing IL-17A and IL-22 in the peripheral blood of SSc individuals

**Figure 1:**
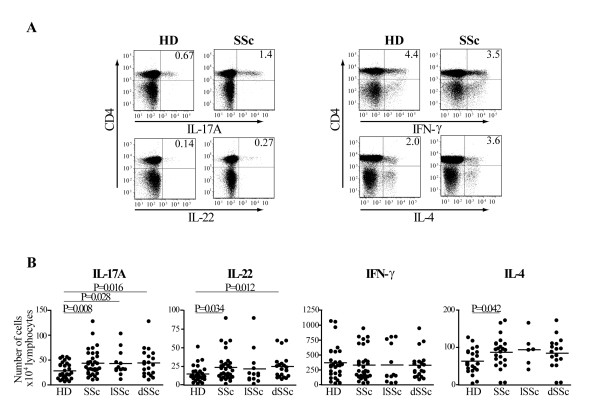
**The number of IL-17A+ and IL-22+ CD4+ T cells is increased in SSc at Day 0**. PBMC were activated by CD3/CD28 crosslinking for 24 h and surface/intracellular stained for FACS analysis. **A**. FACS plots gated on CD4+ T cells for representative healthy donors (HD) and SSc individuals. Numbers in plots indicate the percentage of IL-17A, IL-22, IFN-γ and IL-4 producing CD4+ T cells. **B**. Numbers of IL-17A, IL-22, IFN-γ and IL-4 positive CD4+ T cells for 10^4 ^living lymphocytes in 29 HD and 33 SSc (IL-17A, IL-22, IFN-γ) or 22 HD and 23 SSc (IL-4) individuals. Shown are significant differences assessed by unpaired t-test. lSSc, limited cutaneous SSc and dSSc, diffuse cutaneous SSc.

Several observations support the hypothesis that T cells are responsible for the altered phenotype of fibroblast and endothelial cells, which ultimately leads to fibrosis in SSc individuals [4,5,31]. We aimed at assessing whether in the peripheral blood of a cohort of individuals affected by SSc not receiving disease-modifying drugs, cytokine blocking reagents or immunosuppressant agents, we could detect a peculiar Th cell subset profile when compared to age- and sex-matched HD. To assess the functional repertoire of Th cells, PBMC from individuals affected by SSc and from HD were activated by CD3/CD28 crosslinking overnight. We then detected the intracellular production of IL-17A, IL-22, IFN-γ and IL-4 in the CD4+ T cell fraction by multiparametric flow cytometry. Confirming previous data [32,33], the frequency of CD4+ T cells was higher in SSc than in HD (data not shown). SSc individuals had significantly increased numbers of CD4+ T cells producing IL-4 in their lymphocyte population compared to controls as previously shown [34], while having similar numbers of IFN-γ producing cells (Figure 1A, B). In addition, we observed an increased number of IL-17A and, for the first time, of IL-22 producing cells in SSc (Figure 1B). Interestingly, subgroup analysis showed that IL-17A-producing cells were increased in both lSSc and dSSc, while the increase in IL-22-producing cells was observed in dSSc only (Figure 1B). These data indicate that SSc individuals compared to HD have an imbalance in their Th cells populations.

### Preferential expansion of single IL-17A+, single IL-22+ and double IL17A+IL-22+ producing CD4+ T cells in SSc

**Figure 2:**
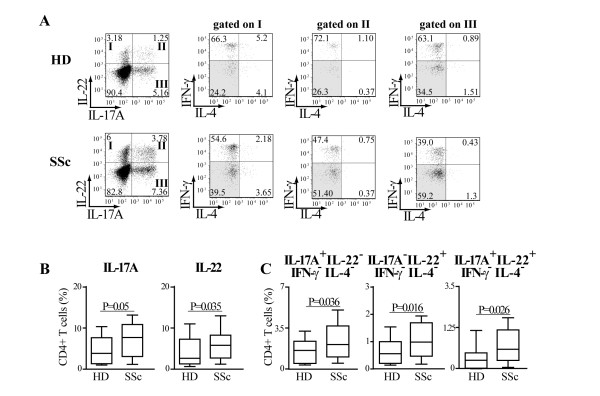
**Th17 and Th22 cells are preferentially expanded in SSc individuals**. PBMC were activated by CD3/CD28 crosslinking and cultured in the presence of IL-2 (20 U/ml). **A**. FACS plots gated on CD4+ T cells of cells harvested at Day 7 of culture and activated by PMA/ionomycin for a representative HD and SSc individual. Intracellular cytokine accumulation was detected by five-color flow cytometry. Numbers in plots indicate the percentage of cells in each quadrant. IFN-γ and IL-4 double negative cells are highlighted by grey shading. **B**. Frequency of IL-17A+ and IL-22+ CD4+ T cells in 29 HD and 30 SSc individuals detected by flow cytometry. **C**. Frequency of CD4+ T cells producing IL-17A alone (IL-17A+IL-22-IFN-γ-IL-4-cells), IL-22 alone (IL-17A-IL-22-IFN-γ-IL-4-cells), and IL-17A in combination with IL-22 (IL-17A-IL-22-IFN-γ-IL-4-cells) in 24 HD and 29 SSc individuals detected by multiparameter flow cytometry analysis.

**Figure 3:**
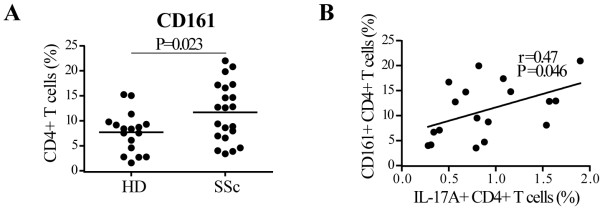
**CD161+ CD4+ T cells are increased in SSc and correlated with the percentage of IL-17A+CD4+ T cells**. **A**, CD4+CD161+ T cells in the peripheral blood of SSc individuals (*n *= 21) and HD (*n *= 17) were identified by two-color flow-cytometry analysis. Bars show the means. **B**, Correlation between the percentage of IL-17A+CD4+ T cells and CD161+CD4+ T cells analyzed *ex vivo*. For cytokine intracellular localization, the conditions were as described in the legend of Figure 2. A two-tailed unpaired *t*-test (A) and Pearson correlation (B) were used for statistical analysis.

Since Th1, Th17 and Th22 cells can all produce IL-22 [35], we investigated whether in SSc individuals IL-22 was produced by the same or by distinct Th subsets. To better discriminate these low frequency subsets, PBMC were expanded *in vitro *for seven days before intracellular cytokine determination. Consistently with the results obtained with overnight cultures, the frequency of cells producing IL-22 was higher in SSc compared to HD after seven-day culture. This was also true for IL-17A (Figure 2A, B). Among all possible cytokine combinations, SSc individuals had a distinct increased frequency of IL-22 single positive (IL-17A-IL-22+IFNγ-IL4-cells) which identify the Th22 subset, and of IL-17A+IL-22+ double positive cells characteristic of Th17 cells. In addition, IL-17A single positive cells were also increased in SSc compared to HD (Figure 2A, C). To substantiate such findings, we tested whether the frequency of CD4+ T cells bearing the CD161 antigen was increased in SSc, since it has been shown that CD161 is preferentially expressed in Th17 cells [21]. This was indeed the case (Figure 3A) and, interestingly, CD161 expression correlated with IL-17A (Pearson r = 0.47, *P *= 0.046) but not IL-22 (Pearson r = 0.34, *P *= 0.17) production in SSc (Figure 3Band data not shown). Of note, CD161 was also expressed in non CD4+ cells; however, these cells were not increased in SSc and were not correlated with the production of IL-17A (not shown).

Altogether our data indicate a preferential expansion of IL-22-single, IL-17A-single and IL-17A/IL-22 double producing CD4+ T cells in SSc, thus supporting the contention that Th22 cells in addition to Th17 cells are expanded in SSc.

### CCR6 expression correlates with the frequency of IL-17 and IL-22-producing cells in SSc but not in HD

**Figure 4:**
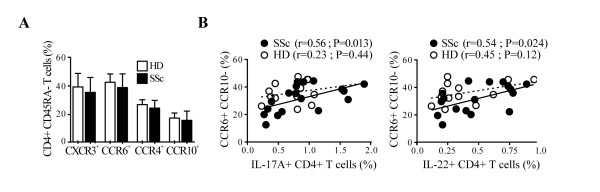
**IL-17A and IL-22 production correlates with CCR6+CCR10- expression in SSc CD4+ T cells**. **A**. Frequency of CD4+CD45RA- T cells expressing CXCR3, CCR6, CCR4 or CCR10 in SSc and in HD detected by a six-color flow cytometry. Columns represent mean ± SD. **B**. Correlation between the percentages of IL-17+ CD4+ T cells or IL-22+ CD4+ T cells with CCR6+CCR10- in CD4+CD45RA- T cells. Full and empty dots represent SSc and HD, respectively. Continuous and dotted regression lines correspond to SSc and HD, respectively. Pearson correlation was used for statistical analysis.

We then investigated whether SSc individuals had a skewed pattern of lymphocytes expressing chemokine-receptors involved in skin- (CCR6, CCR4 and CCR10) [10,12] and lung-homing (CCR6) [11,13,36]. CXCR3, preferentially expressed on Th1 cells, served as control. The expression of CCR6, CCR4, CCR10 as well as CXCR3 on resting peripheral blood memory CD4+ T cells was similar in SSc and HD (Figure 4A). However, CCR6 expression in CD4+ T cells was positively correlated with the frequency of IL-17A+ and IL-22+ CD4+ T cells assessed after overnight activation (not shown). Of note, this positive association was observed in SSc but not in HD and was stronger with the CCR6+CCR10- CD4+ T cell subset for both IL-17A+ (r = 0.56, *P *= 0.013) and IL-22+ (r = 0.54, *P *= 0.024) CD4+ T cells (Figure 4B). No significant positive correlations were observed for the other Th subsets with the chemokine receptors studied. Together, these data suggest that IL-17A and IL-22-producing cells in the peripheral blood of SSc but not HD have skin and lung-homing properties.

### The frequency of IL-22 producing CD4+ T cells distinguishes SSc individuals according to presence of interstitial lung disease

**Figure 5:**
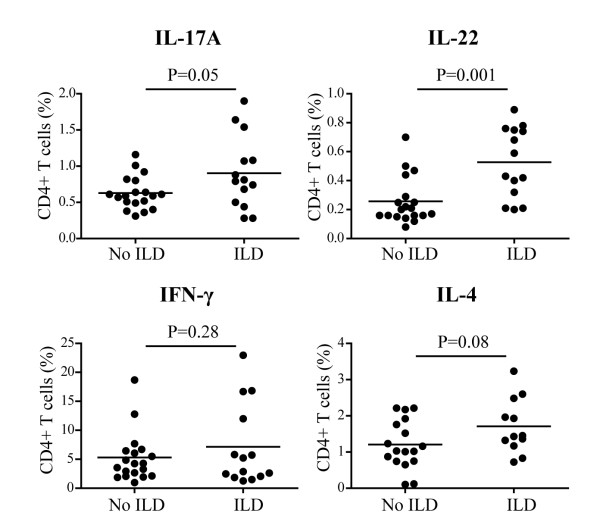
**Increased frequency of IL-22 producing cells in SSc individuals presenting with interstitial lung disease**. Frequency of IL-17A, IL-22, IFN-γ and IL-4 producing CD4+ T cells in SSc individuals presenting or not with ILD. A two-tailed, unpaired *t*-test was used for statistical analysis.

We finally tested whether the production of specific T cell cytokines after overnight activation was associated with SSc clinical characteristics. SSc individuals presenting with ILD, as detected by HRCT and decreased TLC, had higher frequencies of IL-22 (*P *= 0.001) and IL-17 producing T-cells (*P *= 0.05) (Figure 5) when compared with those without ILD. Moreover, IL-4 producing T-cells showed a trend to increase in SSc individuals having ILD (*P *= 0.08), while IFN-γ producing T-cells did not (Figure 5). Of interest, no differences were observed in the frequencies of cytokine producing cells when considering the gender, lSSc vs. dSSc, disease duration, presence of digital ulcers or PAH, and ANA positivity thus reinforcing the specificity of our finding.

## Discussion

In the present study, we demonstrate that Th22 and Th17 cells are specifically increased in the peripheral blood of individuals affected by SSc compared to HD. Moreover, the number of IL-17A and IL-22-producing T cells correlated with CCR6 expression in SSc and not in HD, consistent with enhanced skin- and lung-homing properties for these cells under inflammatory conditions. In addition, we identified a strong relationship between high numbers of IL-22 producing T-cells and SSc ILD.

The relatively low number of individuals included limits the power of our study. In addition, we did not have data on disease activity and quantitative evaluation of the skin involvement. However, our cohort was prospectively recruited and the patients included were not receiving disease-modifying drugs, cytokine blocking reagents or immunosuppressant agents at the time of sampling, which strengthen the reliability of our findings.

By applying a multi-parameter cytofluorimetric analysis we found an increased number of CD4+ T cells producing IL-17A and IL-22 in SSc. They were identified as *bona fide *Th17 and Th22 cells, since single IL-17A+ cells (IL-17A+IL-22-IFNγ-IL4-), single IL-22+ cells (IL-17A-IL-22+IFNγ-IL4-), in addition to double IL-17A+IL-22+ cells (IL-17A+IL-22+IFNγ-IL4-) were distinctly increased in SSc upon seven days of culture. Although unlikely, we cannot exclude that a small percentage of NKT cells or γ/δ T cells co-expressing CD4 could contribute to IL-17 and IL-22 production in our culture system.

Noteworthy, in SSc, and not in HD, the IL-22 and IL-17A T cell numbers strongly correlated with the expression of CCR6, particularly in the absence of CCR10, which may indicate that these cells are prone to be recruited into inflamed target tissues, specifically in SSc, including the lung. Furthermore, we show for the first time that CD4+ T cells expressing the lectin receptor CD161 are increased in SSc and positively correlate with the number of Th17 cells [20].

Our study is the first to assess the presence and functional characteristics of Th22 cells in SSc. It is interesting to note that Th22 cells appear to play important roles in inflammatory skin disorders. For instance, the frequency of IL-22+ T cells in skin derived T-cell lines from psoriasis, atopic eczema and allergic contact dermatitis was significantly higher than in the peripheral blood [17,37-39]. Furthermore, supernatants of skin-derived Th22 clones from psoriatic lesions enhanced wound healing in an *in vitro *injury model, and transcriptome profiling of epithelial cells submitted to the influence of these clones revealed up-regulation of genes involved in tissue remodeling, angiogenesis and fibrosis [37]. In our cohort of SSc, we found that SSc individuals presenting with ILD had increased numbers of IL-22 producing cells. The literature is controversial on the possible mechanisms underlying this association. For instance, lung inflammation was ameliorated in IL-22-deficient mice receiving high doses of bleomycin compared to IL-22-sufficient mice [40]. On the other hand, protection mediated by IL-22 produced by gamma/delta T cells has been reported in a mouse model of lung fibrosis induced by hypersensitivity to *Bacillus subtilis *[41]. While it remains to be established whether the participation of Th22 cells in SSc pathogenic events is harmful rather than protective, their association with ILD suggests an important function of these cells.

Consistent with our findings, IL-17 was previously shown to be increased in the serum and the bronchoalveolar lavage fluid of SSc individuals [27,28]. In addition, an increase in Th17 cells in the peripheral blood of SSc was recently reported [29]. What could be the contribution of IL-17 to SSc pathogenesis remains at the moment speculative. IL-17A has been reported to induce IL-6 and IL-8 production and inter-cellular adhesion molecule 1 (ICAM-1) expression in human fibroblasts [42]. It should be noticed that IL-17A has been shown to participate in an IL-1-dependent manner to the development of bleomycin-induced mouse lung fibrosis [43] and IL-17 may directly stimulate collagen synthesis in rodent fibroblasts [44,45]. However, Kurasawa and colleagues could not demonstrate an increased synthesis of type I and III collagen mRNA in human dermal fibroblasts activated by IL-17 [27]. The different responses of mouse and human fibroblasts to IL-17 may be explained by species-specific characteristics. Thus, our data are in line with the hypothesis that Th17 cells in SSc could be more related to inflammation, autoimmunity, and possibly the generation of autoantibodies [46], as it is speculated for several autoimmune disorders non-characterized by fibrosis, including systemic lupus erythematosus and rheumatoid arthritis in which Th17 cells are increased [26,47,48].

## Conclusions

SSc individuals have an increased frequency of circulating IL-22 and IL-17A, producing cells with skin- and lung-homing potential. The association we found between increased numbers of IL-22-producing cells and ILD indicate that Th22 may act as biomarkers to identify individuals at risk and need to be assessed in larger prospective cohorts of patients.

## Abbreviations

ANA: antinuclear antibody; dSSc: diffuse systemic sclerosis; FCS: fetal calf serum; HD: healthy donor; HRCT: high-resolution computerized tomography; IL: interleukin; ILD: interstitial lung disease; IFN-γ: interferon gamma; lSSc: limited systemic sclerosis; mAb: monoclonal antibody; PAH: pulmonary arterial hypertension; PBMC: peripheral blood mononuclear cell; PMA: phorbol myristate acetate; SLE: systemic lupus erythematosus; SSc: systemic sclerosis; Th: T helper; TLC: total lung capacity.

## Competing interests

The authors declare that they have no competing interests.

## Authors' contributions

MET and NCB conceived the experiments, performed research, analyzed the data and drafted the manuscript. EM performed research. YA provided samples from SSc individuals and critically revised the manuscript. CC conceived research, analyzed the data and drafted the manuscript. All authors read and approved the final manuscript.
